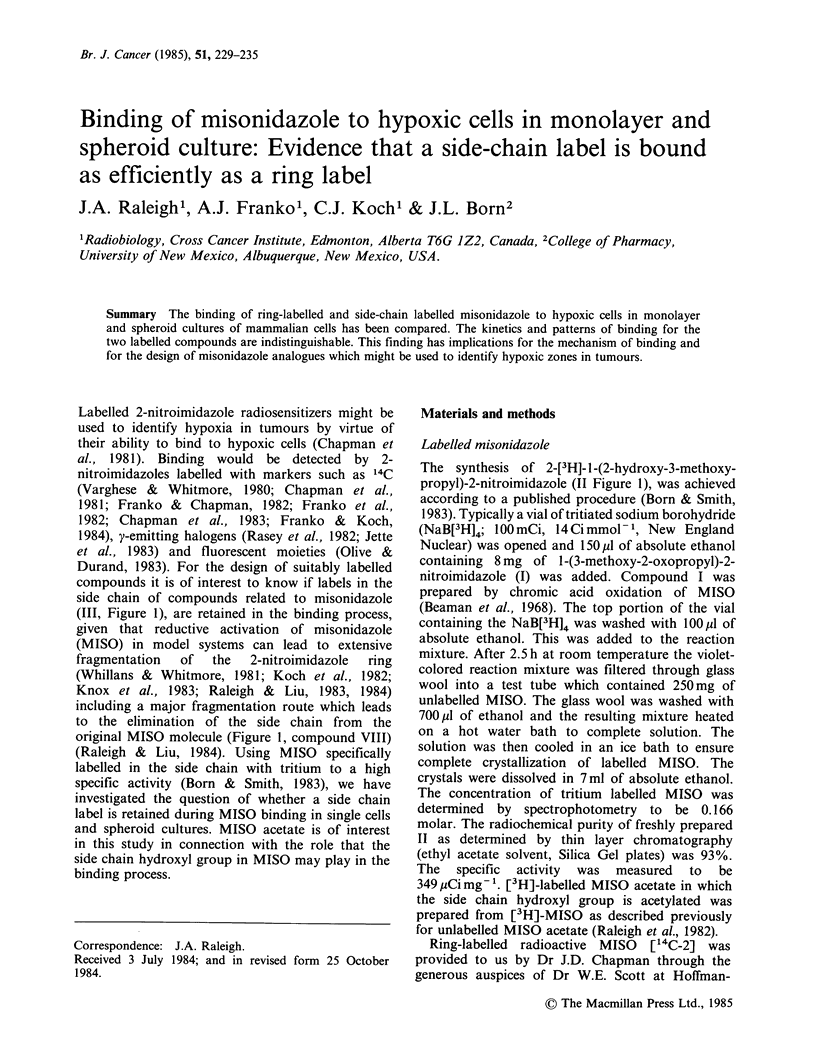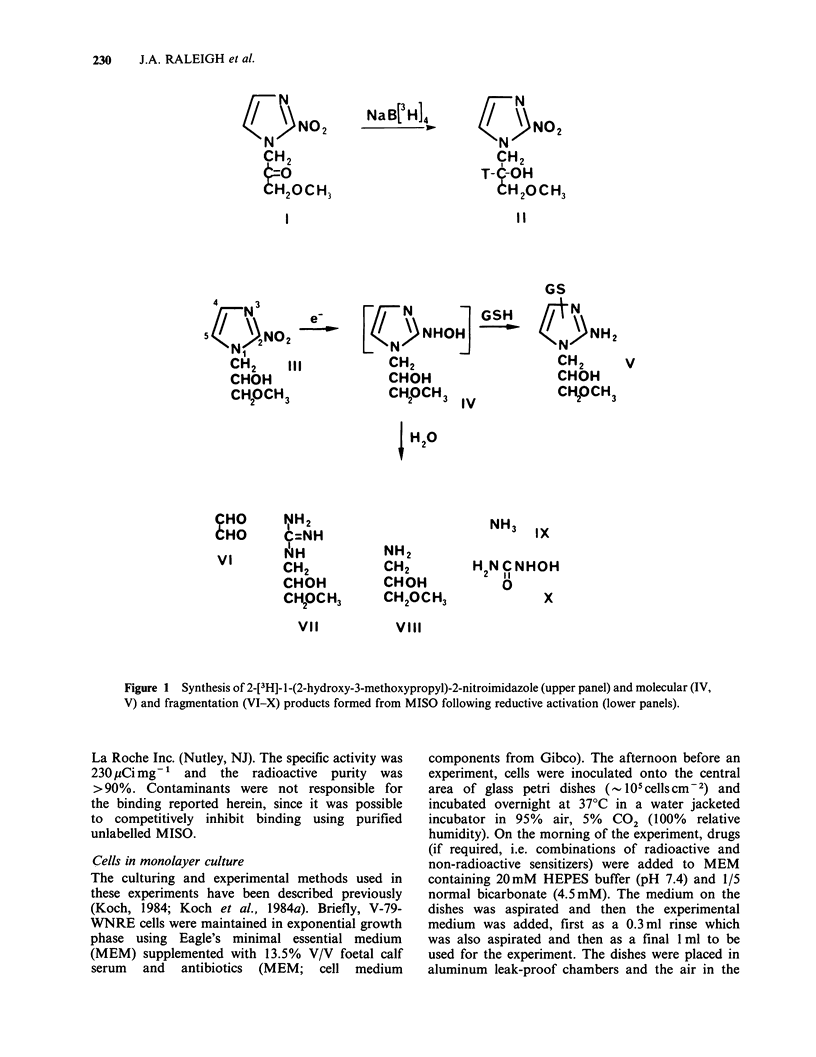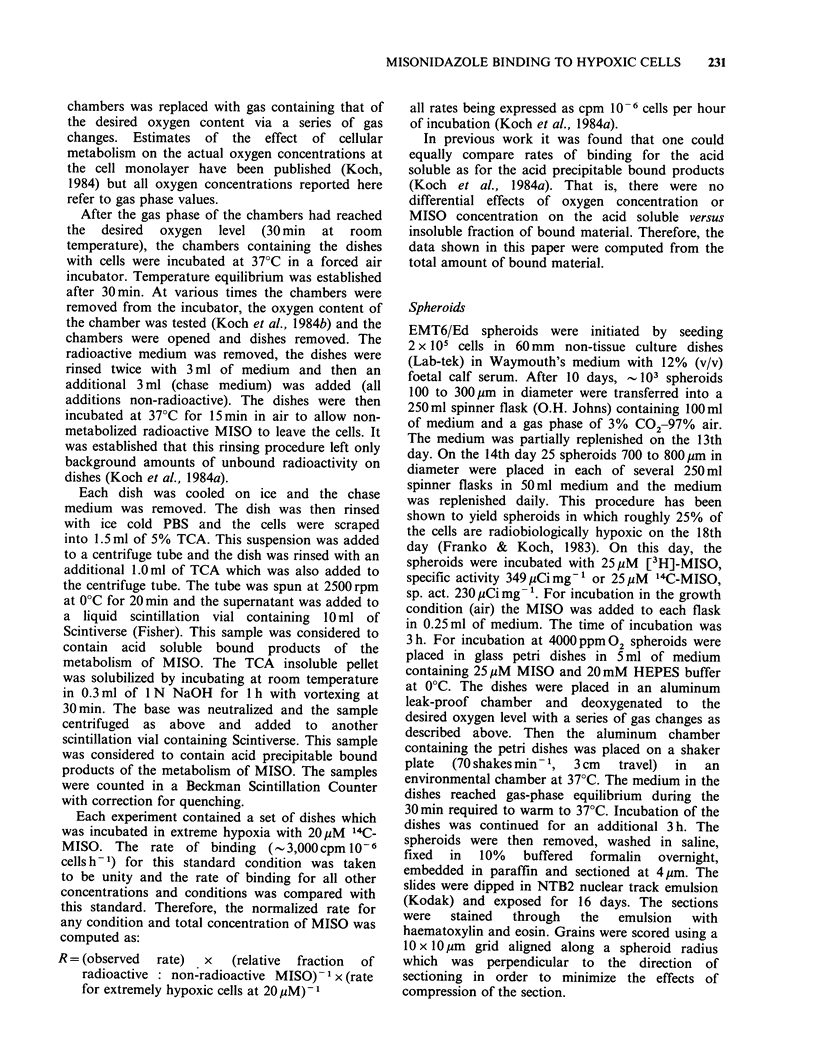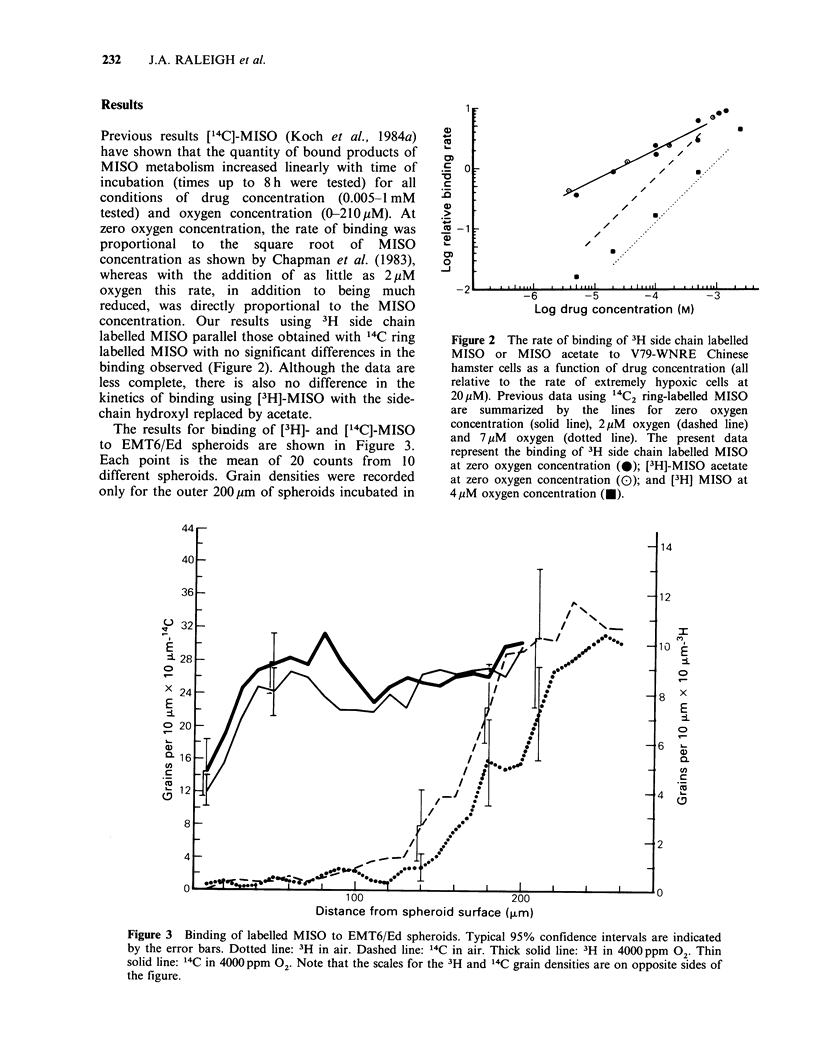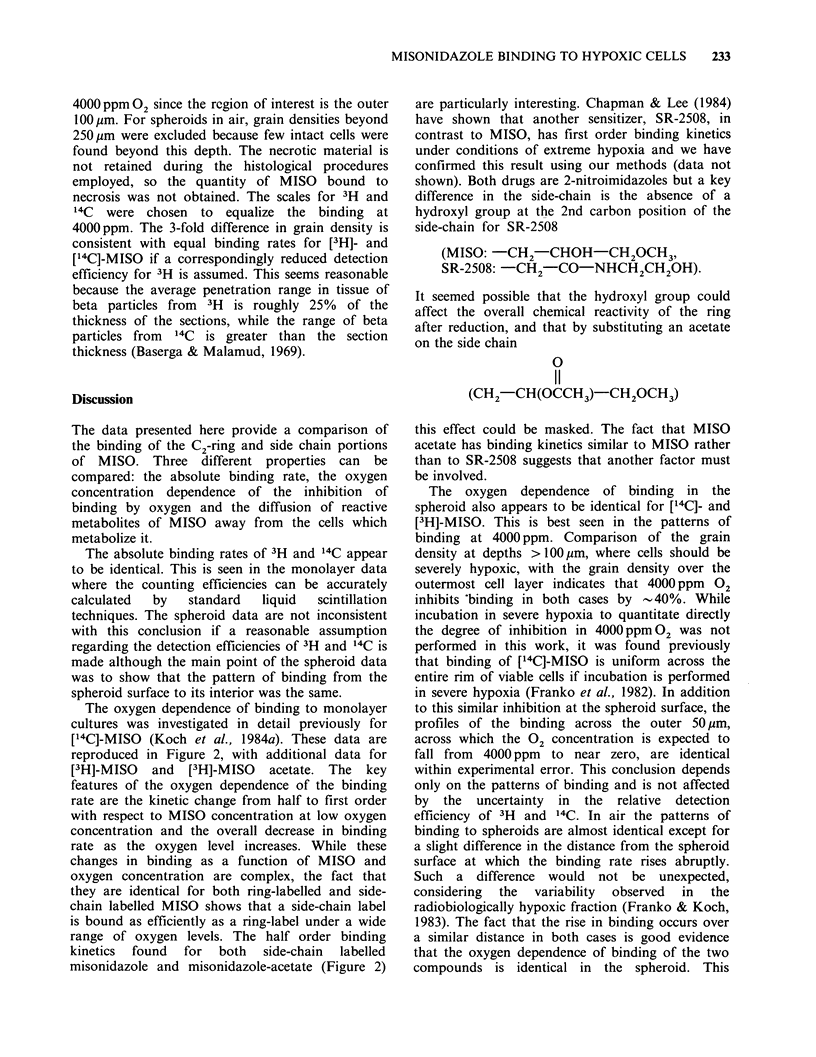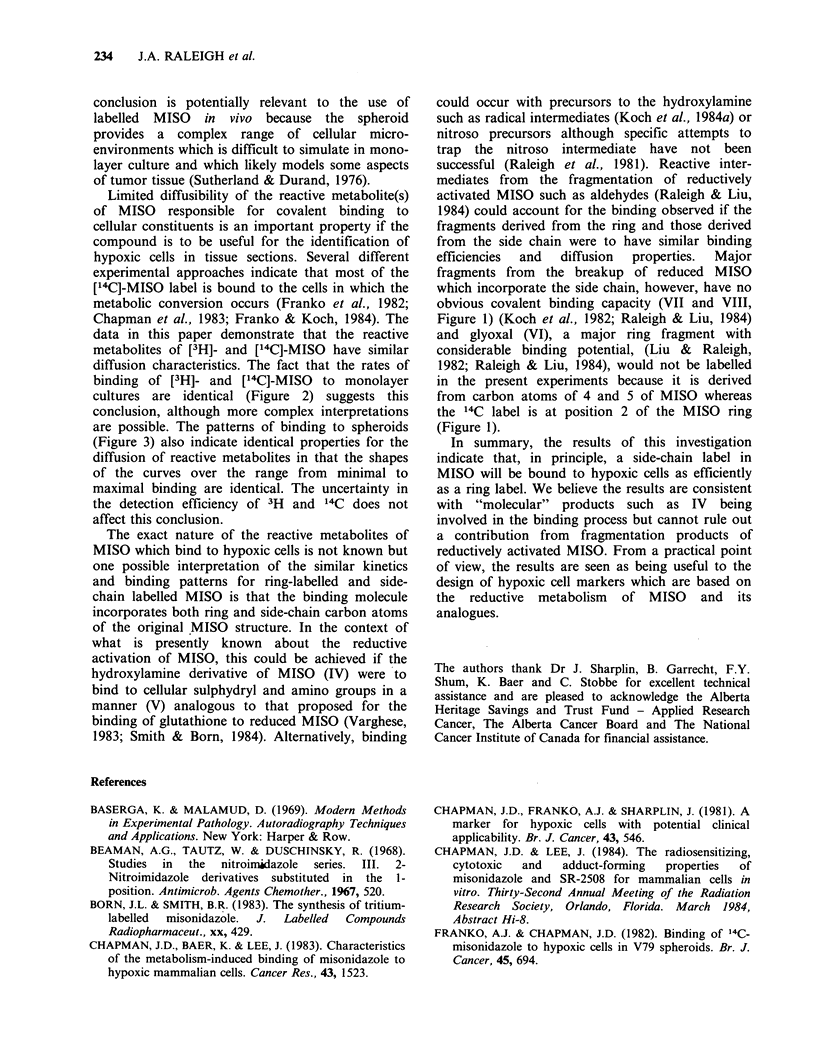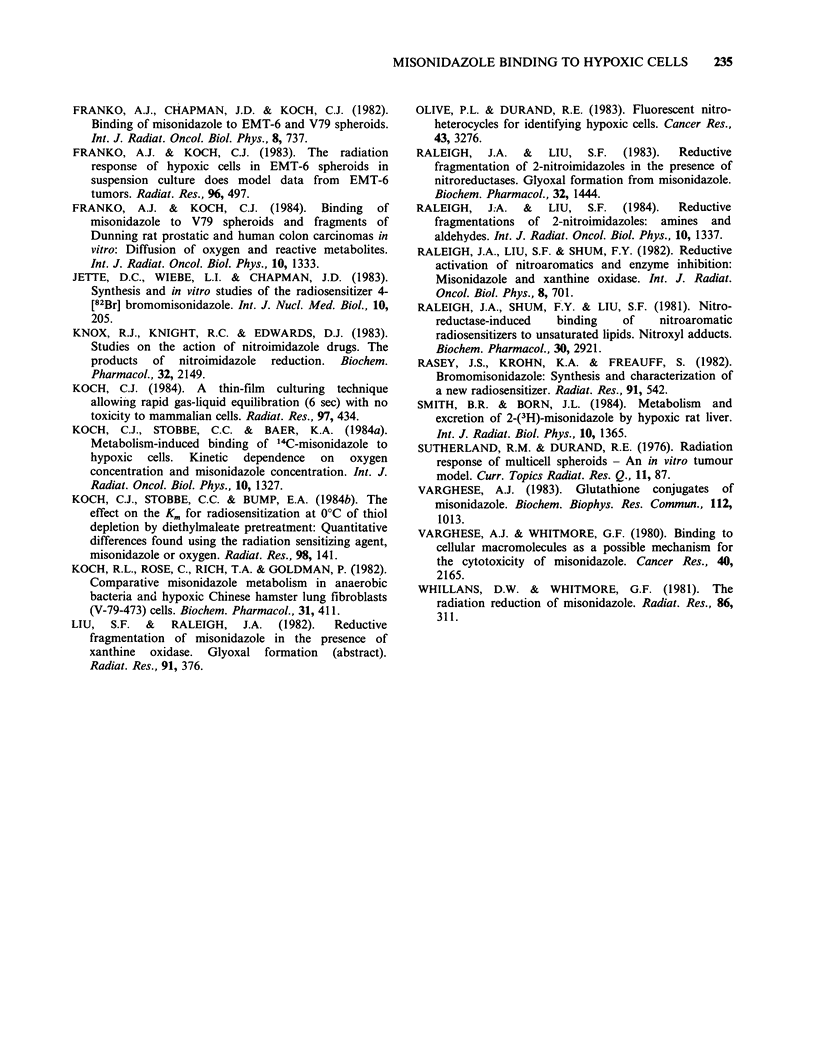# Binding of misonidazole to hypoxic cells in monolayer and spheroid culture: evidence that a side-chain label is bound as efficiently as a ring label.

**DOI:** 10.1038/bjc.1985.33

**Published:** 1985-02

**Authors:** J. A. Raleigh, A. J. Franko, C. J. Koch, J. L. Born

## Abstract

The binding of ring-labelled and side-chain labelled misonidazole to hypoxic cells in monolayer and spheroid cultures of mammalian cells has been compared. The kinetics and patterns of binding for the two labelled compounds are indistinguishable. This finding has implications for the mechanism of binding and for the design of misonidazole analogues which might be used to identify hypoxic zones in tumours.


*Br. J. Cancer* (1985), **51**, 229–235

### Binding of misonidazole to hypoxic cells in monolayer and spheroid culture: Evidence that a side-chain label is bound as efficiently as a ring label

J.A. Raleigh[1], A.J. Franko[1], C.J. Koch[1] & J.L. Born[2]

[1]*Radiobiology, Cross Cancer Institute, Edmonton, Alberta T6G 1Z2, Canada,* [2]*College of Pharmacy, University of New Mexico, Albuquerque, New Mexico, USA.*

**Summary** The binding of ring-labelled and side-chain labelled misonidazole to hypoxic cells in monolayer and spheroid cultures of mammalian cells has been compared. The kinetics and patterns of binding for the two labelled compounds are indistinguishable. This finding has implications for the mechanism of binding and for the design of misonidazole analogues which might be used to identify hypoxic zones in tumours.

Labelled 2-nitroimidazole radiosensitizers might be used to identify hypoxia in tumours by virtue of their ability to bind to hypoxic cells (Chapman *et al.*, 1981). Binding would be detected by 2-nitroimidazoles labelled with markers such as $^{14}C$ (Varghese & Whitmore, 1980; Chapman *et al.*, 1981; Franko & Chapman, 1982; Franko *et al.*, 1982; Chapman *et al.*, 1983; Franko & Koch, 1984), $\gamma$-emitting halogens (Rasey *et al.*, 1982; Jette *et al.*, 1983) and fluorescent moieties (Olive & Durand, 1983). For the design of suitably labelled compounds it is of interest to know if labels in the side chain of compounds related to misonidazole (III, Figure 1), are retained in the binding process, given that reductive activation of misonidazole (MISO) in model systems can lead to extensive fragmentation of the 2-nitroimidazole ring (Whillans & Whitmore, 1981; Koch *et al.*, 1982; Knox *et al.*, 1983; Raleigh & Liu, 1983, 1984) including a major fragmentation route which leads to the elimination of the side chain from the original MISO molecule (Figure 1, compound VIII) (Raleigh & Liu, 1984). Using MISO specifically labelled in the side chain with tritium to a high specific activity (Born & Smith, 1983), we have investigated the question of whether a side chain label is retained during MISO binding in single cells and spheroid cultures. MISO acetate is of interest in this study in connection with the role that the side chain hydroxyl group in MISO may play in the binding process.

Correspondence: J.A. Raleigh.
Received 3 July 1984; and in revised form 25 October 1984.

### Materials and methods

### *Labelled misonidazole*

The synthesis of 2-[$^3$H]-1-(2-hydroxy-3-methoxypropyl)-2-nitroimidazole (II Figure 1), was achieved according to a published procedure (Born & Smith, 1983). Typically a vial of tritiated sodium borohydride ($NaB[^3H]_4$; 100 mCi, 14 Ci mmol$^{-1}$, New England Nuclear) was opened and 150 $\mu$l of absolute ethanol containing 8 mg of 1-(3-methoxy-2-oxopropyl)-2-nitroimidazole (I) was added. Compound I was prepared by chromic acid oxidation of MISO (Beaman *et al.*, 1968). The top portion of the vial containing the $NaB[^3H]_4$ was washed with 100 $\mu$l of absolute ethanol. This was added to the reaction mixture. After 2.5 h at room temperature the violet-colored reaction mixture was filtered through glass wool into a test tube which contained 250 mg of unlabelled MISO. The glass wool was washed with 700 $\mu$l of ethanol and the resulting mixture heated on a hot water bath to complete solution. The solution was then cooled in an ice bath to ensure complete crystallization of labelled MISO. The crystals were dissolved in 7 ml of absolute ethanol. The concentration of tritium labelled MISO was determined by spectrophotometry to be 0.166 molar. The radiochemical purity of freshly prepared II as determined by thin layer chromatography (ethyl acetate solvent, Silica Gel plates) was 93%. The specific activity was measured to be 349 $\mu$Ci mg$^{-1}$. [$^3$H]-labelled MISO acetate in which the side chain hydroxyl group is acetylated was prepared from [$^3$H]-MISO as described previously for unlabelled MISO acetate (Raleigh *et al.*, 1982).

Ring-labelled radioactive MISO [$^{14}$C-2] was provided to us by Dr J.D. Chapman through the generous auspices of Dr W.E. Scott at Hoffman-

© The Macmillan Press Ltd., 1985

**Figure 1** Synthesis of 2-[$^3$H]-1-(2-hydroxy-3-methoxypropyl)-2-nitroimidazole (upper panel) and molecular (IV, V) and fragmentation (VI–X) products formed from MISO following reductive activation (lower panels).

La Roche Inc. (Nutley, NJ). The specific activity was 230 $\mu$Ci mg$^{-1}$ and the radioactive purity was >90%. Contaminants were not responsible for the binding reported herein, since it was possible to competitively inhibit binding using purified unlabelled MISO.

*Cells in monolayer culture*

The culturing and experimental methods used in these experiments have been described previously (Koch, 1984; Koch *et al.*, 1984*a*). Briefly, V-79-WNRE cells were maintained in exponential growth phase using Eagle's minimal essential medium (MEM) supplemented with 13.5% V/V foetal calf serum and antibiotics (MEM; cell medium components from Gibco). The afternoon before an experiment, cells were inoculated onto the central area of glass petri dishes ($\sim 10^5$ cells cm$^{-2}$) and incubated overnight at 37°C in a water jacketed incubator in 95% air, 5% $CO_2$ (100% relative humidity). On the morning of the experiment, drugs (if required, i.e. combinations of radioactive and non-radioactive sensitizers) were added to MEM containing 20 mM HEPES buffer (pH 7.4) and 1/5 normal bicarbonate (4.5 mM). The medium on the dishes was aspirated and then the experimental medium was added, first as a 0.3 ml rinse which was also aspirated and then as a final 1 ml to be used for the experiment. The dishes were placed in aluminum leak-proof chambers and the air in the chambers was replaced with gas containing that of the desired oxygen content via a series of gas changes. Estimates of the effect of cellular metabolism on the actual oxygen concentrations at the cell monolayer have been published (Koch, 1984) but all oxygen concentrations reported here refer to gas phase values.

After the gas phase of the chambers had reached the desired oxygen level (30 min at room temperature), the chambers containing the dishes with cells were incubated at 37°C in a forced air incubator. Temperature equilibrium was established after 30 min. At various times the chambers were removed from the incubator, the oxygen content of the chamber was tested (Koch *et al.*, 1984*b*) and the chambers were opened and dishes removed. The radioactive medium was removed, the dishes were rinsed twice with 3 ml of medium and then an additional 3 ml (chase medium) was added (all additions non-radioactive). The dishes were then incubated at 37°C for 15 min in air to allow non-metabolized radioactive MISO to leave the cells. It was established that this rinsing procedure left only background amounts of unbound radioactivity on dishes (Koch *et al.*, 1984*a*).

Each dish was cooled on ice and the chase medium was removed. The dish was then rinsed with ice cold PBS and the cells were scraped into 1.5 ml of 5% TCA. This suspension was added to a centrifuge tube and the dish was rinsed with an additional 1.0 ml of TCA which was also added to the centrifuge tube. The tube was spun at 2500 rpm at 0°C for 20 min and the supernatant was added to a liquid scintillation vial containing 10 ml of Scintiverse (Fisher). This sample was considered to contain acid soluble bound products of the metabolism of MISO. The TCA insoluble pellet was solubilized by incubating at room temperature in 0.3 ml of 1 N NaOH for 1 h with vortexing at 30 min. The base was neutralized and the sample centrifuged as above and added to another scintillation vial containing Scintiverse. This sample was considered to contain acid precipitable bound products of the metabolism of MISO. The samples were counted in a Beckman Scintillation Counter with correction for quenching.

Each experiment contained a set of dishes which was incubated in extreme hypoxia with 20 $\mu$M $^{14}$C-MISO. The rate of binding ($\sim$3,000 cpm $10^{-6}$ cells $h^{-1}$) for this standard condition was taken to be unity and the rate of binding for all other concentrations and conditions was compared with this standard. Therefore, the normalized rate for any condition and total concentration of MISO was computed as:

$R$ = (observed rate) $\times$ (relative fraction of radioactive : non-radioactive MISO)$^{-1}$ $\times$ (rate for extremely hypoxic cells at 20 $\mu$M)$^{-1}$

all rates being expressed as cpm $10^{-6}$ cells per hour of incubation (Koch *et al.*, 1984*a*).

In previous work it was found that one could equally compare rates of binding for the acid soluble as for the acid precipitable bound products (Koch *et al.*, 1984*a*). That is, there were no differential effects of oxygen concentration or MISO concentration on the acid soluble *versus* insoluble fraction of bound material. Therefore, the data shown in this paper were computed from the total amount of bound material.

### *Spheroids*

EMT6/Ed spheroids were initiated by seeding $2 \times 10^5$ cells in 60 mm non-tissue culture dishes (Lab-tek) in Waymouth's medium with 12% (v/v) foetal calf serum. After 10 days, $\sim 10^3$ spheroids 100 to 300 $\mu$m in diameter were transferred into a 250 ml spinner flask (O.H. Johns) containing 100 ml of medium and a gas phase of 3% $CO_2$–97% air. The medium was partially replenished on the 13th day. On the 14th day 25 spheroids 700 to 800 $\mu$m in diameter were placed in each of several 250 ml spinner flasks in 50 ml medium and the medium was replenished daily. This procedure has been shown to yield spheroids in which roughly 25% of the cells are radiobiologically hypoxic on the 18th day (Franko & Koch, 1983). On this day, the spheroids were incubated with 25 $\mu$M [$^3$H]-MISO, specific activity 349 $\mu$Ci mg$^{-1}$ or 25 $\mu$M $^{14}$C-MISO, sp. act. 230 $\mu$Ci mg$^{-1}$. For incubation in the growth condition (air) the MISO was added to each flask in 0.25 ml of medium. The time of incubation was 3 h. For incubation at 4000 ppm $O_2$ spheroids were placed in glass petri dishes in 5 ml of medium containing 25 $\mu$M MISO and 20 mM HEPES buffer at 0°C. The dishes were placed in an aluminum leak-proof chamber and deoxygenated to the desired oxygen level with a series of gas changes as described above. Then the aluminum chamber containing the petri dishes was placed on a shaker plate (70 shakes min$^{-1}$, 3 cm travel) in an environmental chamber at 37°C. The medium in the dishes reached gas-phase equilibrium during the 30 min required to warm to 37°C. Incubation of the dishes was continued for an additional 3 h. The spheroids were then removed, washed in saline, fixed in 10% buffered formalin overnight, embedded in paraffin and sectioned at 4 $\mu$m. The slides were dipped in NTB2 nuclear track emulsion (Kodak) and exposed for 16 days. The sections were stained through the emulsion with haematoxylin and eosin. Grains were scored using a $10 \times 10$ $\mu$m grid aligned along a spheroid radius which was perpendicular to the direction of sectioning in order to minimize the effects of compression of the section.

### Results

Previous results [$^{14}$C]-MISO (Koch *et al.*, 1984*a*) have shown that the quantity of bound products of MISO metabolism increased linearly with time of incubation (times up to 8 h were tested) for all conditions of drug concentration (0.005–1 mM tested) and oxygen concentration (0–210 $\mu$M). At zero oxygen concentration, the rate of binding was proportional to the square root of MISO concentration as shown by Chapman *et al.* (1983), whereas with the addition of as little as 2 $\mu$M oxygen this rate, in addition to being much reduced, was directly proportional to the MISO concentration. Our results using $^{3}$H side chain labelled MISO parallel those obtained with $^{14}$C ring labelled MISO with no significant differences in the binding observed (Figure 2). Although the data are less complete, there is also no difference in the kinetics of binding using [$^{3}$H]-MISO with the side-chain hydroxyl replaced by acetate.

The results for binding of [$^{3}$H]- and [$^{14}$C]-MISO to EMT6/Ed spheroids are shown in Figure 3. Each point is the mean of 20 counts from 10 different spheroids. Grain densities were recorded only for the outer 200 $\mu$m of spheroids incubated in

**Figure 2** The rate of binding of $^{3}$H side chain labelled MISO or MISO acetate to V79-WNRE Chinese hamster cells as a function of drug concentration (all relative to the rate of extremely hypoxic cells at 20 $\mu$M). Previous data using $^{14}C_2$ ring-labelled MISO are summarized by the lines for zero oxygen concentration (solid line), 2 $\mu$M oxygen (dashed line) and 7 $\mu$M oxygen (dotted line). The present data represent the binding of $^{3}$H side chain labelled MISO at zero oxygen concentration (●); [$^{3}$H]-MISO acetate at zero oxygen concentration (⊙); and [$^{3}$H] MISO at 4 $\mu$M oxygen concentration (■).

**Figure 3** Binding of labelled MISO to EMT6/Ed spheroids. Typical 95% confidence intervals are indicated by the error bars. Dotted line: $^{3}$H in air. Dashed line: $^{14}$C in air. Thick solid line: $^{3}$H in 4000 ppm $O_2$. Thin solid line: $^{14}$C in 4000 ppm $O_2$. Note that the scales for the $^{3}$H and $^{14}$C grain densities are on opposite sides of the figure.

4000 ppm $O_2$ since the region of interest is the outer 100 $\mu$m. For spheroids in air, grain densities beyond 250 $\mu$m were excluded because few intact cells were found beyond this depth. The necrotic material is not retained during the histological procedures employed, so the quantity of MISO bound to necrosis was not obtained. The scales for $^3$H and $^{14}$C were chosen to equalize the binding at 4000 ppm. The 3-fold difference in grain density is consistent with equal binding rates for [$^3$H]- and [$^{14}$C]-MISO if a correspondingly reduced detection efficiency for $^3$H is assumed. This seems reasonable because the average penetration range in tissue of beta particles from $^3$H is roughly 25% of the thickness of the sections, while the range of beta particles from $^{14}$C is greater than the section thickness (Baserga & Malamud, 1969).

### Discussion

The data presented here provide a comparison of the binding of the $C_2$-ring and side chain portions of MISO. Three different properties can be compared: the absolute binding rate, the oxygen concentration dependence of the inhibition of binding by oxygen and the diffusion of reactive metabolites of MISO away from the cells which metabolize it.

The absolute binding rates of $^3$H and $^{14}$C appear to be identical. This is seen in the monolayer data where the counting efficiencies can be accurately calculated by standard liquid scintillation techniques. The spheroid data are not inconsistent with this conclusion if a reasonable assumption regarding the detection efficiencies of $^3$H and $^{14}$C is made although the main point of the spheroid data was to show that the pattern of binding from the spheroid surface to its interior was the same.

The oxygen dependence of binding to monolayer cultures was investigated in detail previously for [$^{14}$C]-MISO (Koch *et al.*, 1984*a*). These data are reproduced in Figure 2, with additional data for [$^3$H]-MISO and [$^3$H]-MISO acetate. The key features of the oxygen dependence of the binding rate are the kinetic change from half to first order with respect to MISO concentration at low oxygen concentration and the overall decrease in binding rate as the oxygen level increases. While these changes in binding as a function of MISO and oxygen concentration are complex, the fact that they are identical for both ring-labelled and side-chain labelled MISO shows that a side-chain label is bound as efficiently as a ring-label under a wide range of oxygen levels. The half order binding kinetics found for both side-chain labelled misonidazole and misonidazole-acetate (Figure 2) are particularly interesting. Chapman & Lee (1984) have shown that another sensitizer, SR-2508, in contrast to MISO, has first order binding kinetics under conditions of extreme hypoxia and we have confirmed this result using our methods (data not shown). Both drugs are 2-nitroimidazoles but a key difference in the side-chain is the absence of a hydroxyl group at the 2nd carbon position of the side-chain for SR-2508

$$(\text{MISO: }—CH_2—CHOH—CH_2OCH_3,$$
$$\text{SR-2508: }—CH_2—CO—NHCH_2CH_2OH).$$

It seemed possible that the hydroxyl group could affect the overall chemical reactivity of the ring after reduction, and that by substituting an acetate on the side chain

$$(CH_2—CH(O\overset{\overset{\displaystyle O}{\|}}{C}CH_3)—CH_2OCH_3)$$

this effect could be masked. The fact that MISO acetate has binding kinetics similar to MISO rather than to SR-2508 suggests that another factor must be involved.

The oxygen dependence of binding in the spheroid also appears to be identical for [$^{14}$C]- and [$^3$H]-MISO. This is best seen in the patterns of binding at 4000 ppm. Comparison of the grain density at depths $>100\,\mu$m, where cells should be severely hypoxic, with the grain density over the outermost cell layer indicates that 4000 ppm $O_2$ inhibits binding in both cases by $\sim$40%. While incubation in severe hypoxia to quantitate directly the degree of inhibition in 4000 ppm $O_2$ was not performed in this work, it was found previously that binding of [$^{14}$C]-MISO is uniform across the entire rim of viable cells if incubation is performed in severe hypoxia (Franko *et al.*, 1982). In addition to this similar inhibition at the spheroid surface, the profiles of the binding across the outer 50 $\mu$m, across which the $O_2$ concentration is expected to fall from 4000 ppm to near zero, are identical within experimental error. This conclusion depends only on the patterns of binding and is not affected by the uncertainty in the relative detection efficiency of $^3$H and $^{14}$C. In air the patterns of binding to spheroids are almost identical except for a slight difference in the distance from the spheroid surface at which the binding rate rises abruptly. Such a difference would not be unexpected, considering the variability observed in the radiobiologically hypoxic fraction (Franko & Koch, 1983). The fact that the rise in binding occurs over a similar distance in both cases is good evidence that the oxygen dependence of binding of the two compounds is identical in the spheroid. This conclusion is potentially relevant to the use of labelled MISO *in vivo* because the spheroid provides a complex range of cellular micro-environments which is difficult to simulate in mono-layer culture and which likely models some aspects of tumor tissue (Sutherland & Durand, 1976).

Limited diffusibility of the reactive metabolite(s) of MISO responsible for covalent binding to cellular constituents is an important property if the compound is to be useful for the identification of hypoxic cells in tissue sections. Several different experimental approaches indicate that most of the [$^{14}$C]-MISO label is bound to the cells in which the metabolic conversion occurs (Franko *et al.*, 1982; Chapman *et al.*, 1983; Franko & Koch, 1984). The data in this paper demonstrate that the reactive metabolites of [$^{3}$H]- and [$^{14}$C]-MISO have similar diffusion characteristics. The fact that the rates of binding of [$^{3}$H]- and [$^{14}$C]-MISO to monolayer cultures are identical (Figure 2) suggests this conclusion, although more complex interpretations are possible. The patterns of binding to spheroids (Figure 3) also indicate identical properties for the diffusion of reactive metabolites in that the shapes of the curves over the range from minimal to maximal binding are identical. The uncertainty in the detection efficiency of $^{3}$H and $^{14}$C does not affect this conclusion.

The exact nature of the reactive metabolites of MISO which bind to hypoxic cells is not known but one possible interpretation of the similar kinetics and binding patterns for ring-labelled and side-chain labelled MISO is that the binding molecule incorporates both ring and side-chain carbon atoms of the original MISO structure. In the context of what is presently known about the reductive activation of MISO, this could be achieved if the hydroxylamine derivative of MISO (IV) were to bind to cellular sulphydryl and amino groups in a manner (V) analogous to that proposed for the binding of glutathione to reduced MISO (Varghese, 1983; Smith & Born, 1984). Alternatively, binding could occur with precursors to the hydroxylamine such as radical intermediates (Koch *et al.*, 1984*a*) or nitroso precursors although specific attempts to trap the nitroso intermediate have not been successful (Raleigh *et al.*, 1981). Reactive intermediates from the fragmentation of reductively activated MISO such as aldehydes (Raleigh & Liu, 1984) could account for the binding observed if the fragments derived from the ring and those derived from the side chain were to have similar binding efficiencies and diffusion properties. Major fragments from the breakup of reduced MISO which incorporate the side chain, however, have no obvious covalent binding capacity (VII and VIII, Figure 1) (Koch *et al.*, 1982; Raleigh & Liu, 1984) and glyoxal (VI), a major ring fragment with considerable binding potential, (Liu & Raleigh, 1982; Raleigh & Liu, 1984), would not be labelled in the present experiments because it is derived from carbon atoms of 4 and 5 of MISO whereas the $^{14}$C label is at position 2 of the MISO ring (Figure 1).

In summary, the results of this investigation indicate that, in principle, a side-chain label in MISO will be bound to hypoxic cells as efficiently as a ring label. We believe the results are consistent with "molecular" products such as IV being involved in the binding process but cannot rule out a contribution from fragmentation products of reductively activated MISO. From a practical point of view, the results are seen as being useful to the design of hypoxic cell markers which are based on the reductive metabolism of MISO and its analogues.

The authors thank Dr J. Sharplin, B. Garrecht, F.Y. Shum, K. Baer and C. Stobbe for excellent technical assistance and are pleased to acknowledge the Alberta Heritage Savings and Trust Fund – Applied Research Cancer, The Alberta Cancer Board and The National Cancer Institute of Canada for financial assistance.

### References

BASERGA, K. & MALAMUD, D. (1969). *Modern Methods in Experimental Pathology. Autoradiography Techniques and Applications*. New York: Harper & Row.

BEAMAN, A.G., TAUTZ, W. & DUSCHINSKY, R. (1968). Studies in the nitroimidazole series. III. 2-Nitroimidazole derivatives substituted in the 1-position. *Antimicrob. Agents Chemother.*, **1967**, 520.

BORN, J.L. & SMITH, B.R. (1983). The synthesis of tritium-labelled misonidazole. *J. Labelled Compounds Radiopharmaceut.*, **xx**, 429.

CHAPMAN, J.D., BAER, K. & LEE, J. (1983). Characteristics of the metabolism-induced binding of misonidazole to hypoxic mammalian cells. *Cancer Res.*, **43**, 1523.

CHAPMAN, J.D., FRANKO, A.J. & SHARPLIN, J. (1981). A marker for hypoxic cells with potential clinical applicability. *Br. J. Cancer*, **43**, 546.

CHAPMAN, J.D. & LEE, J. (1984). The radiosensitizing, cytotoxic and adduct-forming properties of misonidazole and SR-2508 for mammalian cells *in vitro. Thirty-Second Annual Meeting of the Radiation Research Society, Orlando, Florida. March 1984, Abstract Hi-8.*

FRANKO, A.J. & CHAPMAN, J.D. (1982). Binding of $^{14}$C-misonidazole to hypoxic cells in V79 spheroids. *Br. J. Cancer*, **45**, 694.

FRANKO, A.J., CHAPMAN, J.D. & KOCH, C.J. (1982). Binding of misonidazole to EMT-6 and V79 spheroids. *Int. J. Radiat. Oncol. Biol. Phys.*, **8**, 737.

FRANKO, A.J. & KOCH, C.J. (1983). The radiation response of hypoxic cells in EMT-6 spheroids in suspension culture does model data from EMT-6 tumors. *Radiat. Res.*, **96**, 497.

FRANKO, A.J. & KOCH, C.J. (1984). Binding of misonidazole to V79 spheroids and fragments of Dunning rat prostatic and human colon carcinomas *in vitro*: Diffusion of oxygen and reactive metabolites. *Int. J. Radiat. Oncol. Biol. Phys.*, **10**, 1333.

JETTE, D.C., WIEBE, L.I. & CHAPMAN, J.D. (1983). Synthesis and *in vitro* studies of the radiosensitizer 4-[$^{82}$Br] bromomisonidazole. *Int. J. Nucl. Med. Biol.*, **10**, 205.

KNOX, R.J., KNIGHT, R.C. & EDWARDS, D.J. (1983). Studies on the action of nitroimidazole drugs. The products of nitroimidazole reduction. *Biochem. Pharmacol.*, **32**, 2149.

KOCH, C.J. (1984). A thin-film culturing technique allowing rapid gas-liquid equilibration (6 sec) with no toxicity to mammalian cells. *Radiat. Res.*, **97**, 434.

KOCH, C.J., STOBBE, C.C. & BAER, K.A. (1984*a*). Metabolism-induced binding of $^{14}$C-misonidazole to hypoxic cells. Kinetic dependence on oxygen concentration and misonidazole concentration. *Int. J. Radiat. Oncol. Biol. Phys.*, **10**, 1327.

KOCH, C.J., STOBBE, C.C. & BUMP, E.A. (1984*b*). The effect on the $K_m$ for radiosensitization at 0°C of thiol depletion by diethylmaleate pretreatment: Quantitative differences found using the radiation sensitizing agent, misonidazole or oxygen. *Radiat. Res.*, **98**, 141.

KOCH, R.L., ROSE, C., RICH, T.A. & GOLDMAN, P. (1982). Comparative misonidazole metabolism in anaerobic bacteria and hypoxic Chinese hamster lung fibroblasts (V-79-473) cells. *Biochem. Pharmacol.*, **31**, 411.

LIU, S.F. & RALEIGH, J.A. (1982). Reductive fragmentation of misonidazole in the presence of xanthine oxidase. Glyoxal formation (abstract). *Radiat. Res.*, **91**, 376.

OLIVE, P.L. & DURAND, R.E. (1983). Fluorescent nitroheterocycles for identifying hypoxic cells. *Cancer Res.*, **43**, 3276.

RALEIGH, J.A. & LIU, S.F. (1983). Reductive fragmentation of 2-nitroimidazoles in the presence of nitroreductases. Glyoxal formation from misonidazole. *Biochem. Pharmacol.*, **32**, 1444.

RALEIGH, J.A. & LIU, S.F. (1984). Reductive fragmentations of 2-nitroimidazoles: amines and aldehydes. *Int. J. Radiat. Oncol. Biol. Phys.*, **10**, 1337.

RALEIGH, J.A., LIU, S.F. & SHUM, F.Y. (1982). Reductive activation of nitroaromatics and enzyme inhibition: Misonidazole and xanthine oxidase. *Int. J. Radiat. Oncol. Biol. Phys.*, **8**, 701.

RALEIGH, J.A., SHUM, F.Y. & LIU, S.F. (1981). Nitroreductase-induced binding of nitroaromatic radiosensitizers to unsaturated lipids. Nitroxyl adducts. *Biochem. Pharmacol.*, **30**, 2921.

RASEY, J.S., KROHN, K.A. & FREAUFF, S. (1982). Bromomisonidazole: Synthesis and characterization of a new radiosensitizer. *Radiat. Res.*, **91**, 542.

SMITH, B.R. & BORN, J.L. (1984). Metabolism and excretion of 2-($^{3}$H)-misonidazole by hypoxic rat liver. *Int. J. Radiat. Biol. Phys.*, **10**, 1365.

SUTHERLAND, R.M. & DURAND, R.E. (1976). Radiation response of multicell spheroids – An *in vitro* tumour model. *Curr. Topics Radiat. Res. Q.*, **11**, 87.

VARGHESE, A.J. (1983). Glutathione conjugates of misonidazole. *Biochem. Biophys. Res. Commun.*, **112**, 1013.

VARGHESE, A.J. & WHITMORE, G.F. (1980). Binding to cellular macromolecules as a possible mechanism for the cytotoxicity of misonidazole. *Cancer Res.*, **40**, 2165.

WHILLANS, D.W. & WHITMORE, G.F. (1981). The radiation reduction of misonidazole. *Radiat. Res.*, **86**, 311.